# Casting a Wide Net to Fight Coronaviruses

**DOI:** 10.1371/journal.pbio.0030353

**Published:** 2005-09-06

Viewed under a microscope, the coronavirus appears almost beautiful, thanks to the halo-like crown formed by its surface proteins. (“Corona” means “crown” in Latin.) Aesthetics aside, this genus of viruses is responsible for a wide range of animal and human diseases, from the common cold to the deadly severe acute respiratory syndrome, familiarly known as SARS. Research efforts to design antiviral agents to combat coronaviruses intensified after SARS killed at least 800 people in 2003 and have focused mostly on just this virus. But Haitao Yang, Dawei Ma, Zihe Rao, and colleagues reasoned that it might prove more efficient to develop wide-spectrum drugs and vaccines that could work against all coronaviruses—significantly reducing the health and economic burden associated with the 25 species of coronavirus.

Scientists fear that vaccines may prove ineffectual against coronaviruses because the viruses, like HIV, change their protein sequences and structures so often that a vaccine targeting one strain would likely be ineffective against another. The success of such a vaccine strategy depends on finding a protein target that is present, or well conserved, among all the different coronaviruses. By combining structural and biochemical analyses, Yang et al. not only identified such a target in a conserved region of a viral enzyme but also designed compounds with antiviral activity against multiple coronaviruses.[Fig pbio-0030353-g001]

**Figure pbio-0030353-g001:**
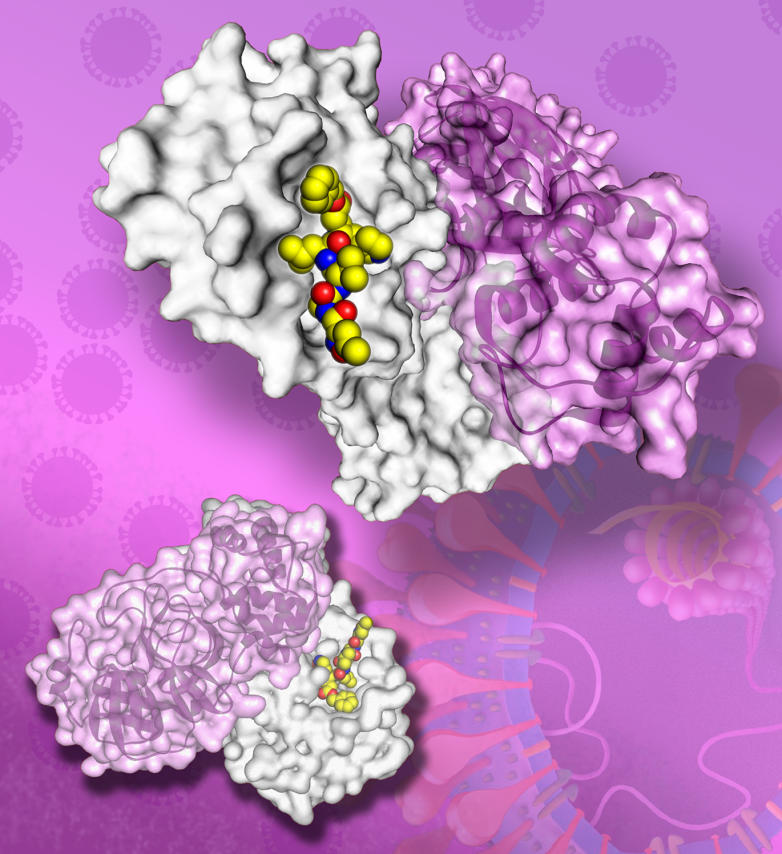
A broad-spectrum inhibitor that can recognize the active site of a coronavirus enzyme called the main protease could lead to the discovery of a single agent against coronaviruses. The protease structure is shown here in ribbon-and-surface representation; inhibitor molecules are the yellow, blue, and red balls

Because coronavirus species show great diversity among their structural proteins—which include the glyocoproteins that form the halo—the authors turned to three enzymes as potential targets. But since structural data were available for only one of the enzymes, called the main protease (Mpro), the authors focused on Mpro. Having structural data in hand greatly accelerates drug development, and since humans and other animals have no proteins similar to Mpro, the likelihood of deleterious side effects is low.

Initial computer analysis showed that the Mpro primary protein sequences (the linear amino acid sequences) have only 38% sequence identity between coronavirus species in some cases. But because three-dimensional structures tend to be more conserved than amino-acid sequences, the authors chose representative viruses from each group of coronavirus to study and compare the structure of their Mpro. This protease normally binds to its target protein (called the substrate) via a specific region, called the substrate-binding site. Structural analysis determined that this site is well conserved among coronaviruses, and biochemical tests confirmed that it would make a promising target for antiviral agents.

To test this hypothesis, the researchers created a synthetic version of the substrate that normally binds to the protease's substrate-binding site—reasoning that if they could inhibit the substrate's access to the binding site by the mimic (known as suicide inhibitors), they should be able to block the protease's activity and maybe halt viral replication. By studying the structure of the protease–substrate/inhibitor complex, Yang et al. continually improved their synthetic inhibitor until it bound strongly to the protease. Using this initial inhibitor as a base, the authors designed a panel of inhibitors and identified compounds that rapidly blocked proteases from multiple coronaviruses and kept the coronaviruses from reproducing. The compounds caused no obvious damage in human cells in the experiments.

The substrate-binding site identified by the researchers is an especially attractive target for drug development because evolutionarily conserved regions do not undergo high mutation rates like the rest of the viral genome, allowing antiviral drugs to maintain their effectiveness. Support for this hypothesis comes from the finding that a compound developed in this study also inhibits Mpro from new coronavirus strains that cause conjunctivitis, bronchiolitis, and pneumonia. By identifying promising candidates for drugs capable of targeting the entire *Coronavirus* genus, Yang et al. have laid the foundation for containing everything from the common cold to the deadly SARS virus. Preclinical and clinical trials will show whether these compounds live up to their promise. —*Supriya Kumar*

